# Influence of Silicon on Biocontrol Strategies to Manage Biotic Stress for Crop Protection, Performance, and Improvement

**DOI:** 10.3390/plants10102163

**Published:** 2021-10-12

**Authors:** Krishan K. Verma, Xiu-Peng Song, Dan-Dan Tian, Dao-Jun Guo, Zhong-Liang Chen, Chang-Song Zhong, Amin Nikpay, Munna Singh, Vishnu D. Rajput, Rupesh Kumar Singh, Tatiana Minkina, Yang-Rui Li

**Affiliations:** 1 Key Laboratory of Sugarcane Biotechnology and Genetic Improvement (Guangxi), Ministry of Agriculture and Rural Affairs/Guangxi Key Laboratory of Sugarcane Genetic Improvement/Sugarcane Research Institute, Guangxi Academy of Agricultural Sciences, Nanning 530007, China; drvermakishan@gmail.com (K.K.V.); xiupengsong@163.com (X.-P.S.); gdj0506@163.com (D.-J.G.); czl_good2007@126.com (Z.-L.C.); 2 Institute of Biotechnology, Guangxi Academy of Agricultural Sciences, Nanning 530007, China; luckytian6688@163.com; 3 College of Agriculture, Guangxi University, Nanning 530004, China; 4 Division of Science and Technology, Guangxi Academy of Agricultural Sciences, Nanning 530007, China; cszhong@126.com; 5 Department of Plant Protection, Sugarcane and By-Products Development Company, Salman Farsi Agro-Industry, Ahwaz 61348-14543, Iran; amin_nikpay@yahoo.com; 6 Department of Botany, University of Lucknow, Lucknow 226007, India; drmunnasingh@yahoo.com; 7 Academy of Biology and Biotechnology, Southern Federal University, 344090 Rostov-on-Don, Russia; rajput.vishnu@gmail.com (V.D.R.); tminkina@mail.ru (T.M.); 8 Centro de Quimica de Vila Real, Universidade de Tras-os-Montes e Alto Douro, Quinta de Prados, 5000-801 Vila Real, Portugal; rupeshbio702@gmail.com

**Keywords:** biotic stress, physio-biochemical/molecular strategies, herbivory, pathogens, plants, silicon

## Abstract

Silicon (Si) has never been acknowledged as a vital nutrient though it confers a crucial role in a variety of plants. Si may usually be expressed more clearly in Si-accumulating plants subjected to biotic stress. It safeguards several plant species from disease. It is considered as a common element in the lithosphere of up to 30% of soils, with most minerals and rocks containing silicon, and is classified as a “significant non-essential” element for plants. Plant roots absorb Si, which is subsequently transferred to the aboveground parts through transpiration stream. The soluble Si in cytosol activates metabolic processes that create jasmonic acid and herbivore-induced organic compounds in plants to extend their defense against biotic stressors. The soluble Si in the plant tissues also attracts natural predators and parasitoids during pest infestation to boost biological control, and it acts as a natural insect repellent. However, so far scientists, policymakers, and farmers have paid little attention to its usage as a pesticide. The recent developments in the era of genomics and metabolomics have opened a new window of knowledge in designing molecular strategies integrated with the role of Si in stress mitigation in plants. Accordingly, the present review summarizes the current status of Si-mediated plant defense against insect, fungal, and bacterial attacks. It was noted that the Si-application quenches biotic stress on a long-term basis, which could be beneficial for ecologically integrated strategy instead of using pesticides in the near future for crop improvement and to enhance productivity.

## 1. Introduction

Silicon, a semi-essential element, induces protection against biotic and abiotic stressors in plants [1,2,3,4]; however, being a major component in soil, it is not yet accepted as a necessary component of plant life. Nearly 95% of the Earth’s crust comprises silica, silicate, or aluminosilicate minerals that contain oxygen, silicon, and aluminum [5]. Silicon has twelve different crystal modifications found in numerous rocks, viz., granite and sandstone.

The change in the pH extends the better availability of macro- and micro-nutrients included in Si fertilizers, which acquire stress resistance in plants. Silicon may increase plant performance, fruit yield, and grain quality [6,7,8,9]. The physiological, biochemical, and molecular responses of plants to biotic and abiotic stressors are remarkably the same when Si is absorbed by the roots and transferred to the shoots by implying its role in defense signaling pathways [4,10]. Soon, the use of Si is expected to become a sustainable strategy with the rising trend in agriculture and horticulture for enhancing crop growth and alleviating abiotic and biotic adverse variables [11,12]. Silicon may help plants to resist pathogen invasion through structural defenses [1,13,14]. It inhibits pathogen colonization by the stimulation of systemic acquired resistance (SAR) and the production of antimicrobial compounds [15,16,17,18] by enhancing the tolerance capacity of plants linked with activation of signaling pathways and expression of genes [4,15,16,19,20].

Plant diseases occurring because of fungal pathogens are one of the most significant restrictions on plant performance [2,7]. Consequently, the use of fungicides and the adoption of tolerance species/ rootstocks were found to be common and more successful methods [21,22] to generate resistance that overcome fungal infections based on genotype–environment interactions [23]. The repeated use of fungicide could cause recurring financial losses and the emergence of disease-resistant populations with severe environmental implications [24,25]. Hence, an alternate eco-friendly approach must be identified in the near future. The disease-suppressing benefits of soil fertilizers with nutritional components with Si have been demonstrated under biotic stress [4,16]. The use of Si has been accepted as a potential alternative over traditional methods due to its crucial role to empower stress tolerance to enhance crop productivity along with agribusiness management [7,15,16,18,26,27]. The various adaptive strategies offered Si-enhanced fungal disease resistance [15,16,18,28] and were found to be linked with significantly higher Si deposits in leaves as a potent physical barrier against the penetration ability of pathogens.

Keeping such unique contributions of Si, such as immunizing any higher biological system against biotic diseases, present in the review are accommodated by recent scientific updates for plant disease resistance influenced by Si through augmentation of antimicrobial chemical synthesis, enzymatic activity, and signaling pathways, which could be useful for researchers in times to come for crop protection and its productivity.

## 2. Availability of Si in Soil

Silicon is the universe’s seventh most plentiful element and the planet’s second most prevalent element. Next to oxygen, Si is the second most abundant compound in the Earth’s crust (~28% weight basis) [2,5,28]. Silicon reacts with oxygen to generate silicates, such as quartz and feldspar. Silicates, viz., quartz and feldspar, are formed when Si combines with oxygen. Silica (quartz) is a silicon and oxygen silicate, whereas feldspars contain other elements besides Si and O_2_. Soils are made up of silicates and alumino-silicate minerals, while rocks are built up from silicates and alumino-silicate compounds. The physiological weathering of silicate releases Si in soil, forming monosilicic acid (H_4_SiO_4_), which does not dissolve at a pH less than 9 and which is absorbed by plant roots from the soil [3,27,29,30,31]. Monosilicic acid concentrations in soil solutions vary from 0.1–0.6 mM [1,32,33]. The growing plants must have a substantial quantity of Si in abundance in soil for its absorption by plants, which is widely distributed in the biological systems [1]. The different types of soils contain varied levels of Si content. For example, in sand, the top strata typically have low concentrations of Si, whereas clay soils include the maximum quantity of phyllosilicates that release Si [34]. Sand comprises quartz (SiO_2_), which has a complicated chemical breakdown strategy, and is more susceptible to Si fertilization than clay soils [35]. An intensive cropping system sequesters Si *ca*. 210–224 mtons from the soil through phytoremediation from cultivated land each year, globally [36,37].

## 3. Action Mechanism of Si and Interaction of Biotic Stress

The action mechanism of Si in plant defense is associated with three actions, viz., the physical, biochemical, and molecular action of mechanisms [22,38], identified as cell wall stiffness reinforcement, papillae formation, callose deposition, signal transduction, and gene expression induced by stressors in plants [4,7,39,40,41].

### 3.1. Silicon Resist Insect Pests’ Diseases

Insects devastate 1/5th of the global agriculture crop yield by consuming leaves, absorbing juice from various plant tissues, and imposing various stresses [42,43] (Table 1). Insect pests may also develop resistance capacity to toxic chemicals in plants [44,45,46]. The herbivorous insects’ host preferences are mainly affected by the physical condition of the host plant, which primarily depends on the nutritional requirements [7,47]. Mineral nutrients such as Si may be added to reduce crop susceptibility to pests [48] to promote insect pest resistance in plants [10,49]. The leaves of *Magnaporthe grisea* having a high accumulation of Si reduces lesion formation [25,50] and improves tolerance capacity in the stem, stalk, and shoot of *Triticum* spp., *Oryza* spp., *Zea* spp., and *Saccharum* spp. against biotic stressors [10,27]. Silicon improves plant resistance to insect damage by developing phytoliths in plant leaves, which increase tissue hardness, weaken herbivore mouthparts, and reduce leaf digestibility (Table 1). It may also impair digestibility by reducing nitrogen and carbohydrate availability during digestion [46,51]. The presence of Si in plant tissue causes metabolic changes that protect the insect pest loss [52]. Generally, insecticides first target the midgut cells of insects [53,54] and cause difficulty in biting and digesting plant tissues due to inert amorphous silica [2,55,56].

A recent study looked at the correlation of feeding Si-containing compounds on the change in the shape of *Tuba absoluta* larvae’s midgut and mandibles [46]. It was also noted that the plants with high Si contents also showed better resistance capacity against *T. absoluta* (Meyrick) attacks. The separation of midgut cells from the basal membrane in *T. absoluta* (Meyrick) caterpillars results in digestive problems [46]. Sucking by insects may be primarily based on a phloem feeder [7,55,56]. The middle lamella’s stiffness and pectin act as a physical barrier to stylet penetration [57]. Silicon deposits would also hamper stylet penetration in cell walls, which are a mechanical barrier. It engaged in the variations of biochemical properties linked to plant defense systems and mechanical constraints against insect harm [22,58]. Silicon alters the structure of trichomes and promotes lignin buildup and the synthesis of phenolics, chitinases, and peroxidases activities, among other defense mechanisms [59]. Many of these characteristics are also linked with plant resistance to sucking insects, which alters their probing behavior [12,28,60].

Unlike folivores, Massey et al. [55] discovered that increasing plant Si did not affect phloem-feeding insects’ feeding population growth performance. This insects’ feeding and secretion of honeydew cause the growth of sooty mold, which reduces *Cucumis sativus* productivity quantitatively and qualitatively [61]. Silicon generates defensive compounds in *C. sativus* [62,63]. Similarly, Si fertilization has a negative impact on green insect preference in *Triticum* spp. [64]. Si-supplied plants may have remarkable higher enzymatic activities. The enhanced activities of CAT and SOD in *Nilaparvata lugen*s (Stl)-infested *Oryza sativa* plants as compared to a control were observed [65].

Silicon-induced resistance to borer caterpillars of the Pyralidae family in *O. sativa* has been observed [66]. However, Si influences the growth of the armyworm (*Spodoptera frugiperda* (Smith) in *Z. mays* [66]. The greenbug (*Schizaphis graminum* Rond) is a severe pest that causes direct and indirect harm by feeding on phloem sap and transmitting viruses and other infections. Silicon showed a negative effect on greenbug eating preferences and lowered reproduction rates in *Sorghum bicolor* and *Triticum* spp. [67,68]. It boosts pathogen defense mechanisms in *Cucumis sativus*. The insect’s feeding, development, longevity, and fecundity were found to be lowered upon Si application [37,69] in the case that it was applied in the soil and combined with one or two foliar sprays [70]. However, the availability of Si in upper plant parts does not necessarily prevent insect herbivory and growth [71].

Silicon fertilization has little influence on *Agrotis ipsilon* survival or mandibular wear [72], with no discernible influence of Si in *Z. mays* on the growth of *Chilo partellus* larvae [73]. Table 1 indicates the impact of Si on different forms of insect pests sucking and chewing on plants. The application of potassium silicate in conjunction with the *Beauveria bassiana* fungus was found to be substantially more effective in killing spider mites (*Tetranychus urticae* Koch), with a casualty rate up to 92% [74]. The signaling molecule (Jasmonic acid) is activated by insect mastication, resulting in the production of herbivore-induced plant volatiles [4,7,75].

Si-amended *C. sativus* attract insect predators, viz., *Dicranolaius bellulus* (Guérin-Méville) against *Helicoverpa armigera* (Hubner) [76]. Soil-applied Si produced n-heptadecane in *Vitis vinifera* infested with *Phalaenoides glycinae* [77]. Similarly, compared to a combination of Si and *B. bassiana*, a single application of Si proved unsuccessful in killing spider mites (*Tetranychus urticae* (Koch)), which led to a significant reduction in mite populations in *Phaseolus vulgaris, Cucumis sativa, Solanum melongena*, and *Zea mays* [25,74]. Another way that Si treatment may help plants against herbivores is by producing hirsute foliage [78] with a buildup of defensive chemicals, viz., phytoalexins, phenolics, and momilactones [58,79,80], and by altering the expression of defense-related genes [4,81,82] as this stimulates jasmonate-mediated defense activities in *O. sativa* plants during insect chewing of *Cnaphalocrocis medinalis* (Guenee) [83,84].

### 3.2. Effects of Silicon on Plant Fungal Diseases

Fungal pathogen-induced diseases are most severe on crop productivity across the globe, qualitatively and quantitatively. Fungal pathogens, viz., *Alternaria solani, Phytophthora infestans, Fusarium oxysporium, Verticilium dahlia*, and *Septoria lycopersici* have been found to be limiting factors for crop productivity and fruit quality [4]. Fungicides and resistant cultivars are the most effective control measures for reducing disease severity [22,85,86]. The use of Si has been promoted as a more promising option for the better management of fungal plant diseases [7,16,23].

Silicon induces a thicker cellulose membrane, while the density of short and long silicified cells in the epidermis of plant leaves, the double cuticular layer, papilla growth, and the thick silica layer beneath the cuticle may help to reduce the severity of illness in plants under field conditions to prevent crop losses [87]. Foliar application of Si extended prevention of powdery mildew in *V. vinifera*, *C. sativus*, and *C. melo* [88,89]. The biogenic Si deposition in *A. thaliana* after activation of callose synthesis [90] often acts as an employed stress signal [91]. The phenolics isolated from Si-applied plants have shown strong fungistatic properties [92]. Cherif et al. [93] found that the Si applied in *C. sativa* plants found higher peroxidase, chitinase, polyphenol oxidases, and -1,3 glucanase activities. Amendment of Si in *Lolium perenne* L. plant had higher chlorogenic acid and flavonoid levels and increased peroxidase and polyphenol oxidase activity in plants infected by *Magnaporthe oryzae*, which are linked with expression of phenylalanine ammonia-lyase and lipoxygenase activities [94].

Silicon-applied plants demonstrated localized cell defense mechanisms through papilla formation, callose synthesis, and the deposition of glycosylated phenolics by *Blumeria graminis*; additionally, changes soil pH to ensure benefits for plants [139,140,141,142,143] to reduce soil and airborne fungal infections (Table 1).

### 3.3. The Impact of Silicon on Plant Bacterial Infections

Silicon has amazing biotic elicitor effects in a variety of plants [52,92,144,145]. Silicon, like commercially available medications such as benzothiadiazole (BTH) and acibenzolar-S-methyl (ASM), has been linked to the development of systemic acquired resistance (SAR) [15]. *Solanum lycopersicum* plant’s resistance to bacterial wilt disease improved by exogenous application of Si [146,147]. Si-applied leaves of *Oryza* spp. [81], *Triticum* spp. [148], and *Cucumis* spp. [148] show an enhancement in an antioxidative enzymatic capacity related to defense mechanisms, resulting in a reduction in disease severity [4,148,149,150], whereas it alters the gene expression in *S. lycopersicum* and *Ralstonia solanacearum* [12,151,152,153,154,155]. The association between Si and the application of biocontrol agents for the better utilization and management of *Podosphaera xantii* (Castagne) in *Cucurbita* spp. [156], yeasts with *Acidovorax citrulli* in *Cucumis melo* [157], and adjuvants for controlling *P. xantii* in *C. melo* [158,159] was found.

Increasing the dose of Si lowered the vulnerability of a cultivar to disease [160] as mangrove plants subjected to extreme climatic circumstances improved their ability to survive [161]. Similarly, deposition of intercellular Si acts as a barrier against disease penetration [162]. Bacterial wilt is widely spread in arid, semi-arid, and temperate regions [163], resulting in partial or complete mortality of *S. lycopersicum* plants [164]. A highly adaptable and varied bacterium, *R. solanacearum*, is the causative agent [165]. Silicon’s involvement as a chemical resistance against the bacterium is significant since it affects quantitative resistance against pathogens (Table 1) [146]. The following are the leading theories for explaining Si-induced tolerance: Si creates chemical compounds that promote plant tolerance and acts as a mechanical barrier against disease advancement [139,151,152,153,166,167]. Kurabachew et al. [167] found that Si and *Bacillus pumilis* greatly reduce the incidence of bacterial wilt by 50% and 27%, respectively. Hence, Si treatment improves plant resistance to bacterial infections.

## 4. Silicon Increase Resistance Mechanism

Despite several research findings on silicon’s effects on fungal infections, its characteristics, efficacy spectrum, and action method are still unknown [15,16,18,168]. When grown in a controlled hydroponic environment, Si does not influence plant performance [26].

### 4.1. Mechanism Physical Barrier

**Figure 1:**
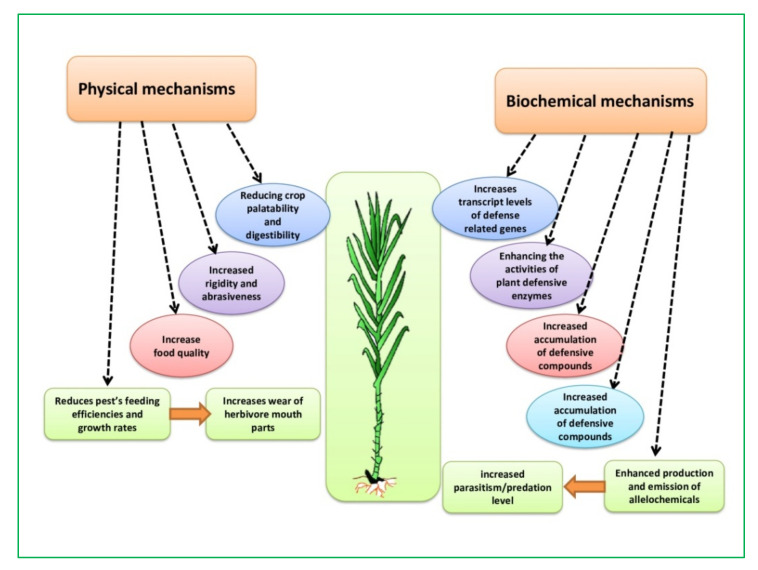
Schematic representation of interaction of silicon with arthropod pests [78].

Silicon accumulated on the surface of tissues acts as a physical disturbance that helps with fungal diseases; according to the first hypothesis, Si improved tolerance capacity. Silicon protects plants from fungal infections by preventing physical penetration, mechanically strengthening plants, and/or making plant cells less vulnerable to pathogen enzymatic breakdown (Figure 1). A thick layer of silica is formed beneath the cuticle of *O. sativa* leaves and sheaths after monosilicic acid polymerization [169]. This Si layer behind the cuticle could be part of what prevents pathogens from penetrating; it could form complexes with organic molecules in the epidermal cell walls, making them more resistant to breakdown by the secretion of enzymes in fungal infections [4,7] and may also be connected to lignin-carbohydrate compounds found in epidermal cell walls [22,170]. Silicified epidermal cell walls were found to be less severe for rice blast disease (*Magnaporthe grisea*) in O. *sativa* [171].

The foliar application of Si causes a physical barrier and osmotic effect in the *Cucumis*–*Podosphaera xanthii* pathosystem. Silicon in the epidermis of Oryza leaves confers resistance to *M. grisea* (blast) appressorial penetration [172]. Heine et al. [173] proposed that the deposition of Si in root cell walls did not act as a physical obstacle to *Pythium aphanidermatum* spreading in *Momordica charantia* and *Solanum lycopersicum* roots. Based on the present findings, it was hypothesized that the tolerance of fungal pathogen in Si-applied plants was considerably more sophisticated than physical tolerance, which has been seriously debated and questioned in recent years [18,25,27,37].

### 4.2. Biochemical Mechanism

**Figure 2:**
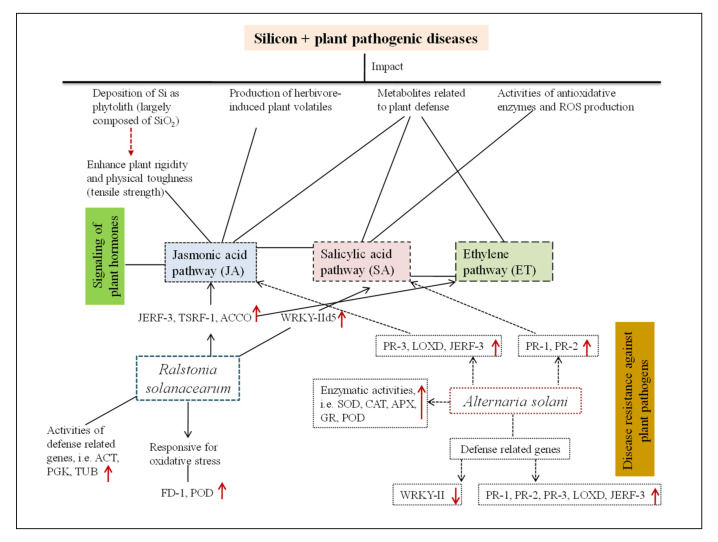
The systematic summary of the Si-mediated regulation of central defense-related genes linked with mitigation of plant pathogenic diseases, i.e., biotic stress. PR-1, PR-2, PR-3—pathogenesis-related proteins; JERF—Jasmonate and ethylene-responsive factor 3; TSRF—tomato stress-responsive factor; ACCO-1—aminocyclopropane-1-carboxylate oxidase; FD-1—ferredoxin-I; POD— peroxidase; WRKY II—WRKY group II transcription factor; LOXD—lipoxygenase; SOD—superoxide dismutase; CAT—catalase; APX—ascorbate peroxidase; GR—glutathione reductase [4,7,41,152]. Up and down arrows indicate the increase or decrease in activity.

Silicon helps plants to defend themselves by increasing different biochemical mechanisms (Figure 1), which boosts antimicrobial enzymes like polyphenol oxidase, glucanase, peroxidase, phenylalanine ammonia-lyase (PAL), phenolics, flavonoids, phytoalexins, and pathogen-related proteins that upregulate various defense signaling pathways such as SA, JA, and ET (Figure 2) [4,7,15,16,17,18] integrated with the induction of various signal transduction pathways [22,25,27]. Salicylic acid activates defense mechanisms primarily against biotrophic and hemibiotrophic pathogens, whereas JA and ET broadly activate defense mechanisms during necrotrophic infections [174]. 

Multiple studies have found that Si regulates plant stress activities by affecting plant hormones’ homeostasis balance and by promoting various signaling pathways [2,78,151,154,175,176,177,178]. Plant hormones accumulate in Si-amended plants in response to pathogen disease and wounding [7,83,176,179]. The plant hormones such as SA, JA, and ET are the first line of protection in increasing the plant responses to different herbivores. JA and SA are associated with defense against herbivores. In particular, JA manages cell-content-feeding and tissue-chewing insects against phloem-feeding-insects [7,180,181].

Due to enhanced production of SA, JA, and ET in *A. thaliana*, plants affected by powdery mildew pathogen in Si-amended *Erysiphe cichoracearum* had greater resistance [176]. Silicon has also been reported to activate the JA and ET signaling pathways in *Solanum lycopersicum* infected with *Ralstonia solanacearum* [151,175,178]. Oryza has been challenged by *Magnaporthe oryzae*, and the impacts of Si on the JA and ET signaling pathways revealed that Si was connected to higher signaling activities, resulting in increased rice tolerance subjected to blast disease [154,177]. In *A. thaliana* powdery mildew disease, Si upregulated the genes’ expression encoding enzymes associated with SA pathway [20,22,27]. According to the Si chemically increased resistance theory, soluble Si in plant organs can be linked to improved fungal disease tolerance. After being infected with necrotizing diseases, many plants developed increased resistance to future pathogen attacks, known as systemic acquired resistance (SAR) [182]. Due to Si application on plants, two mechanisms involved in boosting enzyme activity and antifungal chemical compounds could elicit a defense mechanism comparable to SAR [183], and biochemical and physiological pathways may be implicated in the silicon-mediated disease resistance in plants. 

### 4.3. Role of Defense-Related Enzymes

Silicon enhances disease resistance and delays the growth of invading pathogens by boosting the synthesis of phenolic compounds [184] such as flavonoids, which extend *Rosa* spp. tolerance to *Podosphaera pannosa* and *Triticum* spp. tolerance to *Pyricularia oryzae* [185,186]. Plant defense against pathogen invasion is known to rely heavily on phytoalexins. Silicon treatment boosted phytoalexin production, lowering the incidence of powdery mildew disease caused by *Podosphaera xanthii* in *C. sativa* plants and blast-induced by *M. grisea* in *Oryza* plants [79,187]. Applying Si to cucumber plants increases the production of flavonoid phytoalexins, which protect them from *Podosphaera xanthii* attack [58]. Similar findings have also been discovered in *Oryza* spp., where Si treatment boosts the production of phytoalexins, which enhance blast tolerance activities [79,187]. In perennial ryegrass (*Magnaporthe oryzae)* pathosystems, Si boosts the synthesis of phenolic acids, such as chlorogenic acid and flavonoids, and increases the expression of genes for phenylalanine ammonialyase (PAL) and lipoxygenase, providing tolerance capacity to gray leaf spot disease [94]. Polyphenol oxidase (PPO) has been discovered to play a role in lignin formation to acquire antibacterial effects in host plants [188]. 

Silicon boosts the activity of the enzymes POD and chitinase (CHT), which are important in plant–disease interactions. Peroxidase activity is also associated with cell wall reinforcement and lignin biosynthesis [189], while CHT is one of the primary PR proteins that causes lysis of the cell walls of numerous phytopathogenic fungi [25,37,189,190,191]. In *Cryptococcus-laurentii*–sweet-cherry interactions, enhanced PPO activity reduced infection seriousness in fruits, in the case of applied Si [192]. Pink rot induced by *Trichothecium roseum* has been found to be reduced in melon plants treated with sodium silicate due to increased POD activity [193]. Higher levels of CHT and POD appear to cause increased rice resistance to the brown spot disease (*Bipolaris oryzae*) following Si treatment [194]. The severity of pink rot (*Trichothecium roseum*) has been found to be reduced in sodium silicate-treated Chinese cantaloupe with increased POD and PAL activity [195]. According to Xavier et al. [196], greater CHT and POD activity regulated the improvement in wheat blast tolerance (*Pyricularia oryzae*). In *Phaseolus vulgaris* plants, increased SOD, APX, and GR activities reduced the seriousness of *Colletotrichum lindemuthianum* infection [197]. 

The higher concentration of Si in the aboveground plant parts suffer from various pathogens found to be linked with the most efficient antioxidative metabolic processes (up-regulated APX, CAT, GR, and SOD levels), thereby increasing the removal of ROS production [4,21,198]. Increased activities of PAL, POD, PLO, and CHT in the leaf sheaths of Si-supplied *Oryza* plants led to a reduction in the progression of sheath blight lesions (*R. solani*) [199]. Enhancing the activation of CHT, SOD, POD, and 1,3-glucanase in *Cucumis melo* plants reduced powdery mildew (*Podosphaera xanthii*) [200]. Perennial ryegrass grown in Si-amended soil demonstrated higher POD and PPO activities after *Magnaporthe oryzae* infection [94]. Rice resistance to *Pyricularia oryzae* enhances SOD, CAT, APX, GR, and lipoxygenase activities [198]. 

### 4.4. Genomics and Metabolomics Prospective

In plants, Si is linked to several physiological and biochemical activities, the stimulation of signal pathways, and the augmentation of disease resistance expression of genes with respect to plant–disease interaction [4,15,20]. Studies at the transcriptomic and proteomic levels have demonstrated Si’s defense responses in multiple patho-infections [151,155,176,201]. Silicon boosts the activity of WRKY transcription factor; causes the creation of an infection tolerance response to protein, ferritin, late embryogenesis abundant protein; and increases the activity of trehalose phosphatase, resulting in tomato plant resistance to *Ralstonia solanacearum* [2,7,152]. Similar results were obtained in rhizobacteria-inoculated tomato stems and tomato genotypes treated with Si after inoculation with *R. solanacearum*. A greater proportion of down-regulated expression of genes was also correlated to photosynthetic pathways [167]. Silicon changes cell wall structure, resulting in hypersensitive reactions, hormone synthesis, PR proteins, and antimicrobial compounds (Figure 2) [15,25]. 

The application of Si to *S. lycopersicum* plants inoculated with *R. solanacearum* resulted in a significant enhancement in protein levels, implying that Si mediates disease resistance through a relative shift in protein levels [19]. Silicon works as a pathogen resistance modulator in the host [15,18]. Under optimum conditions, there was no discernible difference in gene expression without Si application [202]. Kauss et al. [203] discovered that the creation of a proline-rich protein paired with the presence of silica near the site of pathogen penetration confers resistance to infection in *C. sativus* plant leaves. Brunings et al. [154] used a microarray to investigate the gene expression of Si-treated *Oryza sativa* and discovered that 221 genes, including some transcription factors (TFs), were differentially regulated compared to the control. Silicon boosted the photorespiration in *Oryza* plant leaves affected by *Cochliobolus miyabeanus* substantially, according to Agilent 44K oligo DNA arrays [204]. According to genome-wide analyses, the significant number of genes associated with host plant defense strategies were differentially expressed and unique in *Solanum lycopersicum*, *Oryza sativa*, *Arabidopsis*, and *Triticum aestivum* plants cultivated in Si-applied soil (Figure 2) [4,22,151,154,155,176,202].

The silicon-dependent microarray approach for expression of genes in *Oryza* spp. was first investigated by Watanabe et al. [202]. The addition of Si increased the level of a zinc finger protein homolog while decreasing the expression of chlorophyll *a/b* binding protein, metallothione-like protein, *Xa*21 gene family member, and carbonic anhydrase homolog [41,202]. Generally, zinc finger proteins act as major TFs for stress-related genes, which may enhance stress resistance capacity in Si-amended plants [41]. Transcription factors are the major regulators of downstream genes necessary for plant resistance to biotic stressors for stress-induced genes. Transcription factors are normally aided by specific cis-elements termed regulons, which are found in the target gene promoter section [41,205,206,207].

Transcription factors’ upregulation in response to Si might interact with cis elements in the promoter area of genes implicated in stress tolerance, triggering stress tolerance to biotic stressors. To protect the plants from stress, regulatory genes may also promote the transcription of genes linked with defense-related or stress-responsive pathways, such as the phenylpropanoid pathway or ABA-dependent or ABA-independent regulatory pathways [41]. Silicon increases the transcript levels of pathogenesis-related genes, i.e., PR1, PR2 (glucanse), PR3 (chitinase), and other TFs, resulting in increased tolerance efficiency to a variety of pathogenic diseases [4]. Genes involved in Si uptake and accumulation have been studied in several plants, including *Hordeum vulgare, Zea mays, Cucurbita pepo, Triticum aestivum, Cucumis sativus*, and *Equisetum arvense* [181,208,209]. The Si-influx transporters *Lsi*1 and *Lsi*6 are members of the aquaporin family and are linked to Si buildup in plant organs [41,210]. In comparison to *Oryza sativa* plants, the Si absorption capability of *Lsi*1 and *Lsi*2 differs substantially in a range of plant varieties [4,181,211].

## 5. Is Si Essential/Beneficial Element?

Japan and Korea were the first countries to understand the relevance of Si in crop yield, particularly in *Oryza* spp., in the 1950s. Researchers in other nations have identified Si as an agronomically important nutrient. In 2004, Brazil became the third country to recognize Si formally. According to the Brazilian Ministry of Agriculture, which regulates commercial fertilizer production, Si is an essential micronutrient. In various countries, sources of Si are only sold as soil amendments or conditioners rather than fertilizers because Si is still not accepted as an important mineral element. It seems to be a necessary plant nutrient based on the different criteria established by Epstein and Bloom [212]. The requirement of Si has been established in the literature for a wide range of plant species, demonstrating the importance of Si for plant health [7,9,31,213,214].

## 6. Conclusions and Future Perspectives

Recent advances have explored Si absorption, transport, and accumulation in higher plants as an element having several beneficial effects. Consequently, Si buildup in plants extend the dynamics of its absorption using specialized transporters. Its availability and accumulation in plants may improve plant performance and productivity during biotic and abiotic stressors under adverse environmental variables. Therefore, a battery of knowledge is yet to be acquired through experimentation by researchers in times to come to reveal and integrate physiological, biochemical, and molecular mechanisms regulated by Si transporters in plants in response to defense against biotic and abiotic stresses, as it seems prudent to consider Si application as normal and costless to upgrade plant performance, productivity, and biomass yield linked with physiological fitness for sustainable agriculture under the era of climate change.

## Figures and Tables

**Table 1:** Effects of silicon on plant pests and related resistance mechanisms.

**Crop**	**Pests**	**Pest Species**	**Adaptive Mechanisms**	**Source**
**Sugarcane** (*Saccharum officinarum* L.)	Stalk borer	*Diatraea saccharalis* (Lepidoptera: Crambidae)	Enhanced Si accumulation and relative growth rate and decreased boring success of sugarcane borer larvae and feeding injury. Upgraded cuticle thickening and crystals accumulation on the leaf stomata.	[95,96,97]
	African stalk borer	*Eldana saccharina* (Lepidoptera: Pyralidae)	Si-amended plants significantly enhanced the accumulation of Si in soil and plant organs relative to normal plants, and the outer rind was harder than the control. Treated plants reduced borer penetration, stalk injury, and gain of larval mass. Si directly supported the resistance of *E. saccharina* through a decreased larval growth rate and feeding injury to the crop plants and indirectly supported it by delayed stalk penetration, resulting mostly in an enhanced exposure time frequency of mature larvae to natural enemies.	[98,99,100]
	Stalk borer	*Sesamia* spp. (Lepidoptera: Noctuidae)	Si increased the tolerance efficiency of sugarcane against stalk borers. The significant loss on borer population and damage but the major loss in the stalk injury (%), bored internodes, moth exit holes, and length of borer tunnel and number of larvae and pupae per 100 stalks were monitored in the sensitive cultivar. It enhanced cane and juice quality parameters and efficiency of parasitism.	[101,102,103]
	Spittlebug	*Mahanarva fimbriolata* (Hem.: Cercopidae)	Si enhanced the uptake, accumulation, and nymphal mortality. It totally depended on the sugarcane cultivars. The duration of pre-oviposition, fecundity, and egg viability were found to be unchanged by Si amendment.	[104]
	Internode borer	*Chilo infuscatellus* (Lepidoptera: Crambidae)	Si reduced the damage incidences and was significantly effective against early shoot borers.	[105]
	Leafhopper	*Pyrilla perpusilla*	The *Pyrilla* population was less in the Si-applied field, and parasitism (%) increased. The *Pyrilla* population reduced by an increment of *E. melanoleuca* parasitism with Si amendment.	[106]
	Yellow mite	*Oligonychus sacchari* (Acari: Tetranychidae)	Significant differences were found in Si and control groups of mite and predatory beetle populations. The population density of mites decreased in all the Si-applied categories as compared to control plants. It is the potential element for the management of mite injury and should be applied with other management approaches.	[107,108]
	Stalk borer	*Diatraea tabernella* (Lepidoptera: Pyralidae)	The amendment of Si-based products decreased internodes borer (about 50%) loss.	[109]
**Rice** (*Oryza sativa* L.)	Asiatic stem borer	*Chilo suppressalis* (Lepidoptera: Crambidae)	Si-applied plants enhanced Si concentration relative to normal plants and reduced borer penetration, weight increase, stem injury, and prolonged penetration time and larval behavior. Plant mortality by stem borer, leaf folder, and population size of the plant hopper were positively reduced. The results showed that the application of Si may provide substantial protective capacity from a few of the rice pests during field conditions.	[110,111]
	Brown planthopper	*Nilaparvata lugens* (Hemiptera: Delphacidae)	The higher dose of Si had no symptoms on the morphological traits. It is the major element that restricts brown planthopper (BPH) response in rice–BPH interactions, and it is more beneficial for non-pesticide BPH control.	[112]
	White-backed planthopper	*Sogatella furcifera* (Hemiptera: Delphacidae)	Increased Si content in the upper and lower sides of rice leaves in the foliar spray of Si. Sufficient Si cells were found around the stomata. The oxalic acid and soluble sugar content were enhanced significantly. The number of eggs laid by per female of *S. furciferafed* was reduced.	[113]
	Yellow stem borer	*Scirpophaga incertulas* (Lepidoptera:Crambidae)	All the soil treatments reduced damage by YSB at vegetative and reproductive phases across five varieties as compared to the control. Si revealed the enhanced deposition of Si in cell walls and a two- to five-fold increase in Si content across treatments. The histological studies showed the rupture of the peritrophic membrane, increased vacuolation, disintegration of columnar cells, and discharge of cellular contents into the gut lumen due to abrasion of midgut epithelium, as compared to the control where the columnar cells and midgut lining were intact.	[114]
**Papaya** (*Carica papaya* L.)	Spotted spider mite	*Tetranychus urticae* (Acari: Tetranychidae)	Plant leaves were performed to investigate the physiological parameters that indicate the activation of the defense strategy of plants. Si induced the formation of plant defense substances decreasing, the net reproduction rate.	[115]
**Tahiti Lime** (Citrus spp.)	Asian Citrus Psyllid	*Diaphorina citri* (Homoptera: Liviidae)	The use of Si in seedlings and trees infected Asian citrus psyllid (ACP) oviposition, causing a loss of about 60%. It did not affect the macro-micro nutrient profile of plants, with the exception of the foliar application.	[116]
**Pepper** (*Capsicum annum* L.)	Chilli Thrips	*Scirtothrips dorsalis* (Thysanoptera: Thripidae)	A very low impact of Si on the leaf morphological injury and numbers of thrips restored from diseased plants were observed. Jasmonic acid as a plant defense elicitor did not change the proportion of the leaves that sustained thrips injury. Plant roots absorb Si in the soil but are not distributed or translocated to the other plant organs, i.e., leaf and shoot. No significant effects were observed in the plant biomass.	[117]
**Strawberry** (Fragaria × ananassa)	Spotted spider mite	*Tetranychus urticae* (Acari: Tetranychidae)	Si prolonged the frequency of some immature phases of the mites in parental and F_1_ generations; no changes were found at the complete biological cycle. The time of pre-oviposition and oviposition and the longevity of the parental generation and the longevity and oviposition of the F_1_ generation of the two-spotted spider mite were negatively affected by the addition of Si.	[118]
*Zinnia elegans*	Aphid	*Myzus persicae* (Hemiptera: Aphididae)	No changes were found at the duration of the pre-reproductive and survivorship of *M. persicae* by Si, but the total cumulative fecundity and the intrinsic rate of increase (r(m)) were slightly decreased on *Z. elegans* plants subjected to Si. Si content increased in plant leaves. Phenolics compounds and guaiacol peroxidase (GPX) activity were slightly affected.	[119]
**Tomato** (*Solanum lycopersicum* L.)	Silver whitefly	*Bemisia tabaci* (Hemiptera: Aleyrodidae)	Si reduced the population of immature whiteflies on tomato plants. Foliar spray was more efficient in decreasing the density of population of these pests as compared to Si irrigation.	[120]
	Leaf miner	*Tuta absoluta* (Lepidoptera: Gelechiidae)	A potential impact of Si on crops for increasing plant vigor and tolerance to pest injury was observed. Si reduced the population of immature tomato leaf miners on tomato crops.	[120]
**Tomato** (*Lycopersicon esculentum* Mill.)	Cotton thrips	*Frankliniella schultzei* (Thysanoptera: Thripidae)	Si enhanced the number of lesions and the mortality of nymphs, reduced the injury on tomato leaves, and increased the tolerance strategy to pests.	[121]
**Collard greens** (*Brassica oleracea*)	Diamond back moth	*Plutella xylostella* (Lepidoptera: Plutellidae)	Nutritional variations mediated by stress and Si in fiber, LWC, soluble N, and glucosinolates did not enhance insect activities in any feeding guild.	[122]
	Cabbage aphid	*Brevicoryne brassicae* (Hemiptera: Aphididae)	Si improved the resistance capacity of stress and herbivore stresses.	[122]
**Soybean** (*Glycine max* L.)	Budworm	*Helicoverpa punctigera* (Lepidoptera: Noctuidae)	Herbivory decreased leaf biomass in Si-applied and normal plants compared to herbivore-free plants. Si and herbivory enhanced the Si level. It decreased *H. punctigera* relative growth rates.	[123]
	Silver whitefly	*Bemisia tabaci* (Hemiptera: Aleyrodidae)	No effects were found on silverleaf whitefly oviposition, but significant mortality in nymphs was found. Si enhanced the resistance degree to silverleaf whitefly and down-regulated the phenolic compounds, but no effect on lignin formation and the vegetative growth phase was observed. However, an enhanced tolerance capacity to plants was observed.	[124]
**Wheat** (*Triticum aestivum* L.)	Pink stem borer	*Sesamia inferens* (Lepidoptera: Noctuidae)	Si enhanced the photosynthetic performance, biomass, and productivity.	[125]
	Aphid	*Schizaphis graminum* (Hemiptera: Aphididae)	The aphid’s intrinsic rate of population increased after seedling emergence and the enzymatic activities, i.e., POD, PPLO, and PAL associated in the plant defense mechanisms.	[64]
	Grain Aphid	*Sitobion avenae* (Hemiptera: Aphididae)	The density of wheat aphids was enhanced during N application, which closely correlates to the losses of the average soluble sugar and total phenolic content. The effects of the Si on the reduction in population density of aphids would be associated to the increment of the average contents of soluble sugar, phenolic compounds, and tannin contents of wheat leaves and ears.	[126]
**Sunflower** (*Helianthus annuus* L.)	Bordered patch	*Chlosyne lacinia* (Lepidoptera: Nymphalidae)	Reduced weight of the caterpillars at the first and second week of age was observed. Si increased the distribution of the element and decreased lignin content. Negative correlations were found in Si and larval weight. It is an alternative strategy that can effectively integrate into the management of pest in crops.	[127]
**Cucumber** *(Cucumis sativa* L.)	Silver whitefly	*Bemisia tabaci* (Hemiptera: Aleyrodidae)	Si-treated plant leaves were less injured as compared to normal plants. No positive signs were found in treated and normal plants regarding lignin content, nutritional elements, water status, trichome density, and carbon and nitrogen levels. Volatile organic compounds and indole content increased for plant defense priming, and cellulose content was reduced.	[62,128]
**Cucumber** (*Cucumis sativus* L.)	Cotton Bollworm	*Helicoverpa armigera* (Lepidoptera: Noctuidae)	Herbivory positively enhanced the accumulation of Si in infected plant leaves. The use of Si upregulated Si and the C:N ratio while reducing the larval relative consumption and the relative growth rate in the in situ assays.	[129]
**Cocoa** (*Toxoptera aurantii*)	Aphid	*Toxoptera aurantii* (Aphididae)	The efficiency of the chlorophyll fluorescence yield of PSII (*Fv/Fm*), photosynthetic responses, and total soluble phenol activities were significantly enhanced. The amendment of Si did not affect the morphological performance index.	[130]
**Bean** (*Phaseolus vulgaris* L.)	Silver whitefly	*Bemisia tabaci* (Hemiptera: Aleyrodidae)	No changes were observed in the oviposition of the whitefly and the nymph development as well as the phenol levels after Si amendment.	[131]
**Bean** (*Phaseolus vulgaris* L.)	Spider mite	*Tetranychus urticae* (Acari: Tetranychidae)	Si suppressed the *T. urticae* egg-laying, population growth, and leaflet damage and slightly mitigated *T. urticae*-induced losses in photosynthetic responses.	[132]
**Potato** (*Solanum tuberosum* L.)	Beetle	*Diabrotica speciosa* (Chrysomelidae)	No significant interactions were found between Si and crop parameters. The incidence of beetles and aphids was not influenced by Si application and neither was the growth, development, and final output of the crop plants.	[133]
**Grape** (*Vitis vinifera* L.)	Grapevine moth	*Phalaenoides glycinae* (Lepidoptera: Noctuidae)	Application of Si may also indirectly affect plant pests through induced chemical defenses by altering and increasing the production of herbivore-induced plant volatiles (HIPVs). It plays a major role in induced plant defense strategies activated by herbivore feeding or oviposition.	[77]
**Maize** (*Zea mays* L.)	Fall armyworm	*Spodoptera frugiperda* (Lepidoptera: Noctuidae)	Si reduced the larval weight, pre-pupal weight, pupal weight and larval survival, and fecundity and fertility. The biological characteristics of *S. frugiperda* were non-significantly correlated with increasing levels of Si, phenols, tannins, and potassium levels in plant leaves.	[134]
	True armyworm	*Pseudeletia unipuncta* (Lepi-doptera: Noctuidae)	Effectively decreased the palatability and digestibility of the plant leaves and thus impacted nutrient uptake by insect herbivores. The addition of Si increased larval mortality as compared to the control because early instars with poorly developed mandibles could not feed effectively.	[135]
**Rescuegrass** (*Bromus catharticus*)	Grasshopper	*Oxya grandis* (Orthoptera: Acrididae)	Si enhanced more than 12 times the higher supplementation treatments. The maximum dose of Si in Si-rich plants did not affect the morphological structure of the phytoliths.	[136]
Sitka spruce (*Picea sitchensis*)	Large pine weevil	*Hylobius abietis* (Coleoptera: Curculionidae)	No significant effects were shown on the growth or mortality of plants after Si application. Bark Si content was found to be similar as compared to normal seedlings.	[137]
**Ryegrass** (*Lolium perenne* L.)	African Armyworm	*Spodoptera exempta* (Lepidoptera: Noctuidae)	Si decreased the digestibility of plant leaves and decreased the functionality with *S. exempta*-ingested food to body mass and the amount of nitrogen absorbed from their food, leading to a decreased rate of insect growth.	[138]
